# Experimental evidence suggests the existence of evolutionary conserved global operation principles governing microbial metabolism

**DOI:** 10.1038/srep03017

**Published:** 2013-10-22

**Authors:** Sergio Bordel

**Affiliations:** 1 Chemical and Biological Engineering Department. Chalmers University of Technology, Kemivägen 10, SE 412 96 Gothemburg, Sweden

## Abstract

The search for optimization principles in microbial metabolism, such as biomass or ATP yields or growth rate optimization, has attracted substantial research efforts in the recent years. Here we use the results of C13 labeling experiments together with genome scale metabolic networks of *S cerevisiae* and *E coli* in order to assess if there are relationships between systemic variables that are present in both organisms. Strong correlations between the total flux per unit of substrate and the ATP turnover rate per unit of substrate and between the growth rate divided by the total flux and the total flux per unit of substrate were observed for both organisms. We also observed that the common assumption of biomass yield optimization is not consistent with the experiments.

The recent development of sequencing technologies and bioinformatics has made possible the reconstruction of genome-scale metabolic networks for different microorganisms^1^. Genome-scale metabolic networks can be condensed into a mathematical representation that, by imposing steady state constraints to internal metabolites and directionality constraints to the metabolic reactions, define a region of feasible flux distributions^2^, which from a geometric point of view is a cone in a multidimensional Euclidean space (with as many dimensions as metabolic reactions). By setting maximal rates for some of the reactions (typically substrate uptake rates), the region of feasible flux distributions is further reduced to a convex polytope in a multidimensional Euclidean space.

Genome-scale metabolic networks contain only stoichiometric information. In order to use these networks as tools to predict in-vivo metabolic flux distributions and how these flux distributions change after genetic manipulations such as gene deletions, other assumptions about the operation principles of metabolism are necessary.

In this paper we use experimental flux distributions measured in two different microorganisms, *S cerevisiae* and *E coli*, with the aim of identifying possible additional criteria (objective functions or extra constrains), that could be used in order to improve the predictive power of genome-scale metabolic models.

Linear objective functions (typically the growth rate) have been extensively used to compute metabolic flux distributions^3^. If a maximal substrate uptake rate is set as a constraint the optimization of the growth rate becomes equivalent to the optimization of the biomass yield. The mentioned yield optimization has shown to generate correct predictions for microorganisms growing in chemostats^4^ for a broad scope of dilution rates and different carbon sources^5^. The common phenomenon of overflow metabolism, in which less energetically efficient fermentative pathways are used in detriment of the more efficient respiration, even if oxygen is available, has been explained in terms of a trade-off effect which favors fast growth in detriment of high yield^6^,^7^. Within the frame of constraint based modeling, this overflow metabolism phenomenon is typically addressed by using an upper bound for the oxygen consumption, which limits the extent of respiration^8^, and maximizing the growth rate. The implicit assumption in this approach is that even if catabolism is not optimal (the less efficient fermentation is partially used instead of the more efficient respiration), anabolism is optimal and the ATP produced in catabolism is optimally allocated to maximize the cell growth. This assumption of optimal anabolism appears experimentally to be correct for microbial cultures growing in chemostats^5^,^9^. Here we test if optimal anabolism works in batch cultures. Growth yield optimization has shown to work poorly for microorganisms in batch cultures^9^. It has been shown that experimental flux distributions observed in the central carbon metabolism of *E. coli* seem to be more consistent with the maximization of ATP production rate per flux unit^8^, however this predictions were done using a metabolic network with only 10 degrees of freedom (while genome-scale networks have typically hundreds of degrees of freedom). In metabolic networks with more degrees of freedom (which can involve futile cycles), the optimization of this function is likely to predict flux distributions in which there is not biomass production and all the ATP produced is degraded in futile cycles. Also based on a metabolic network of *E. coli*'s central carbon metabolism, it has been argued that the experimental flux distributions of *E. coli* and other bacterial species are close to the optimality Pareto surface that defines the trade-offs between maximal biomass yield, maximal ATP production yield and minimal sum of metabolic fluxes^10^. The main problem of assessing the suitability of objective functions by comparing predicted and measured flux distributions is the fact that in order to predict a flux distribution, one or more constraints (at least the uptake rate of a limiting substrate) have to be used together with the tested objective function^9^,^11^ and formally any objective function is potentially able to generate correct predictions, provided that it is combined with a suitable set of constraints.

Another limitation is the fact that the experimental techniques available to measure metabolic flux distributions, which are based on using C13 labeled substrates^12^,^13^,^14^,^15^, are only able to resolve a limited number of flux splitting ratios in the central carbon metabolism. For example, the determined flux ratios measured in one of the datasets that we are using^16^ allow determining 7 independent internal metabolic fluxes, while genome-scale metabolic networks have typically hundreds of degrees of freedom^17^. This means that there are many possible flux distributions consistent with the experimental measurements.

It is a common practice to infer the flux distributions in a larger network from a reduced number of measured fluxes by first computing one solution compatible with the constraints in the larger network in which the set of measured fluxes is as close as possible to the measured values^10^, this is typically done by minimizing a sum of weighted error squares. The larger network has more degrees of freedom than the number of measured fluxes; therefore there are many possible solutions compatible with the values obtained from the minimization of squares. In order to obtain a single solution, the sum of squares of the non-measured fluxes is minimized^10^. The mentioned approach involves 2 main problems. First of all if the large network is supposed to be a genome-scale network, a steady state solution in which all the measured fluxes are in agreement with the measured values (or at least within the error measurement intervals) should exist and the error minimization would not be necessary. If this is not the case whether the large network is not comprehensive enough or the measured values are not correct. On the other hand, by minimizing the sum of squares of the fluxes, an arbitrary assumption is being made and this could bias the results. Here we argue (see methods) from an information theoretical perspective, that the best estimate of a global flux distribution given a limited set of measured fluxes is the average of the components of the convex basis of the space of solutions compatible with the measurements.

## Results

Using available C13 labeling experimental data for two of the best known eukaryotic and prokaryotic microorganisms (*S cerevisiae* and *E coli*), we aim to identify possible global operation principles that govern the metabolic flux distributions and that are general enough to be conserved between two microorganisms that are very far apart from an evolutionary perspective. The genome-scale metabolic models that we used are *iTO980*^4^ for *S cerevisiae* and *iJR904*^18^ for *E coli*. We have the experimental flux distributions of a reference *S. cerevisiae* strain and 36 deletion mutants^16^ as well as the flux distributions for a reference *E coli* strain, 3 deletion mutants and 6 strains evolved from the deletion mutants (2 evolved strains per mutant)^19^. The genome-scale metabolic models were constrained with the reported experimental error intervals^16^,^19^. In all the cases there were feasible solutions within the reported intervals for the measured fluxes. No error minimization was therefore required. A random sampling algorithm^18^ was used to generate a set of 3000 flux distributions. The sampled distributions correspond to elements of the convex basis that define the region of flux distributions consistent with the measurements (See methods for more details). The average flux distributions for each strain are reported in the supplementary files S1 and S2.

For each of the obtained flux distributions, we computed 19 systemic variables such as: the sum of all the metabolic fluxes, biomass yield on the substrate, biomass per unit of total flux, the total flux per unit of consumed substrate, the ATP, NADH and NADPH turnover rates, the previous turnover rates per unit of substrate and per unit of total flux etc. As we have argued in the previous section the actual value of these systemic variables cannot be inferred from experiments (using the current C13 labeling methods), and what we computed are optimal estimators of these variables (See methods).

Here we computed the correlation coefficients between the estimators for the systemic variables in order to identify possible relations of the types: y = a + bx or z = a + bx + cy. Only 3 relations with correlation coefficients higher than 0.8 and conserved both for *S cerevisiae* and *E coli* were observed, these relations corresponded to: a positive correlation with ordinate at the origin equal to zero between the ATP turnover rate and the NADH turnover rate (correlation coefficients of 0.953 for *E coli* and 0.938 for *S cerevisiae*), a positive correlation with negative ordinate a the origin between the total flux per unit of substrate and the ATP turnover rate per unit of substrate (correlation coefficients of 0.879 for *E coli* and 0.980 for *S cerevisiae*) and a negative correlation with positive ordinate at the origin between the growth rate divided by the total flux and the total flux per unit of substrate (correlation coefficients of (0.919 for E coli and 0.909 for S cerevisiae).

**Figure 1:**
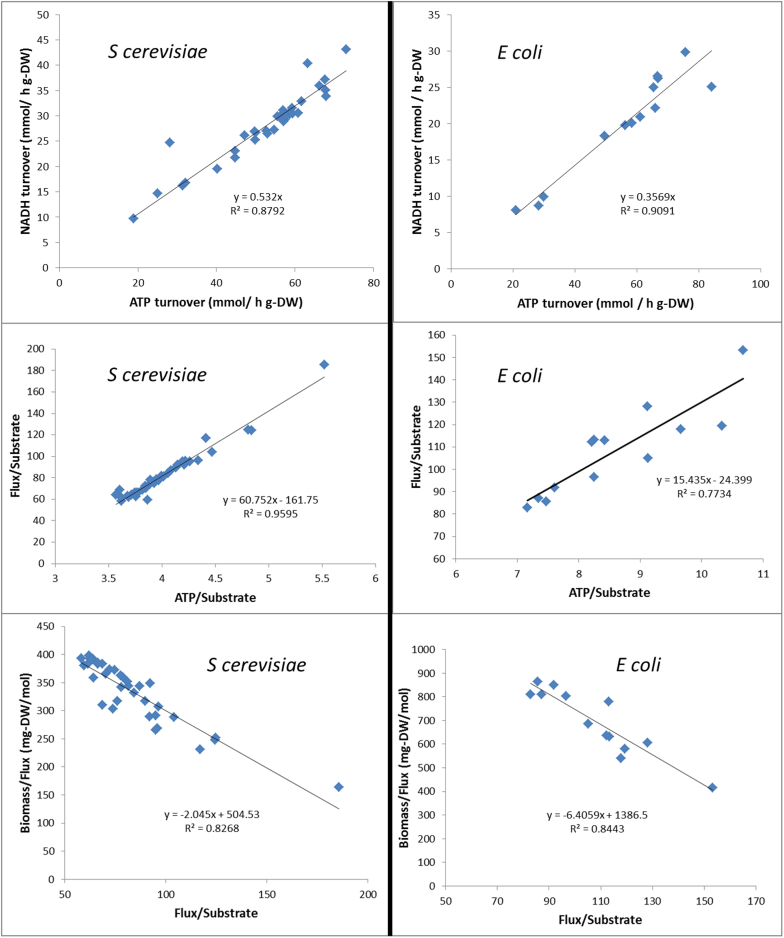
Linear correlations between systemic variables in S. cerevisiae and E. coli.

The slopes and the ordinates at the origin are different for each organism (Figure 1) but the type of relation is conserved. The existence of so conserved correlations in two microbial organisms so far apart from an evolutionary point of view and with very different metabolic networks, seems to indicate the existence of conserved operation principles common to microbial cells, which govern the distribution of metabolic fluxes. These relationships between systemic variables could be used as constraints in genome-scale metabolic models in order to improve their predictive power. The experimental data that we have used correspond to conditions of glucose excess and growth in batch reactors, which as we have mentioned are the conditions in which the common assumption of biomass yield optimization tends to fail.

**Figure 2:**
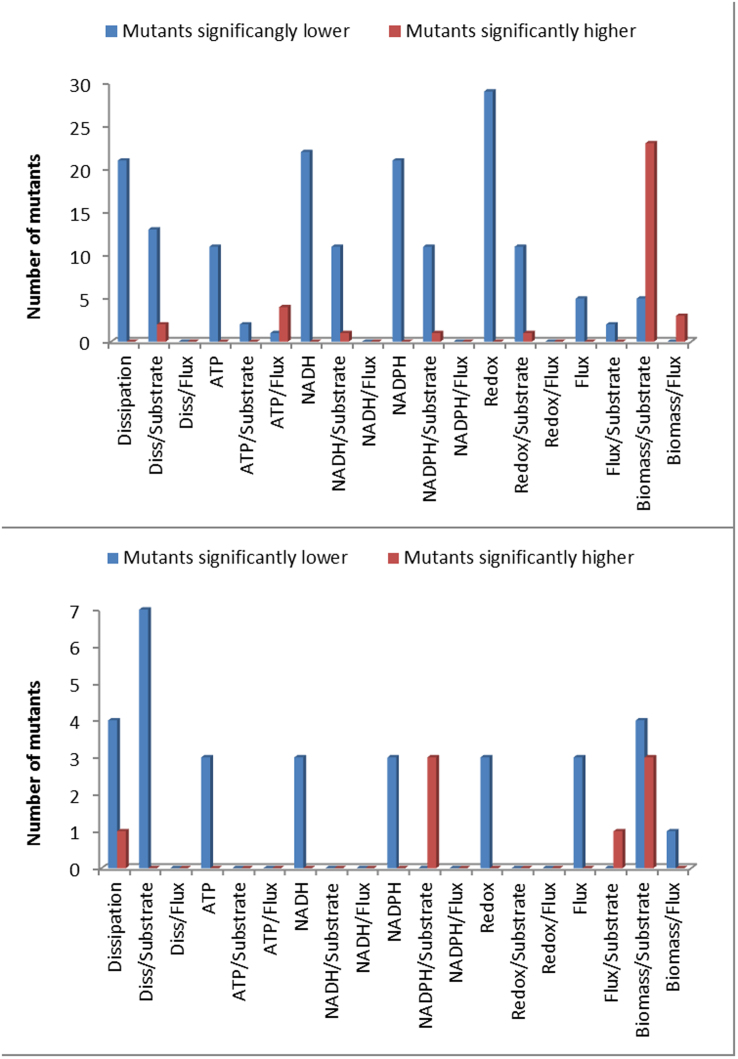
The bars represent the number of deletion mutants (and evolved strains for E. coli) that showed higher or lower values for each systemic function with a probability higher than 95%. The biomass yield shows higher values in many deletion mutants than in the wild type. The turnover rate of redox cofactors seems to be a suitable objective function (maximal in the wild type).

**Figure 3:**
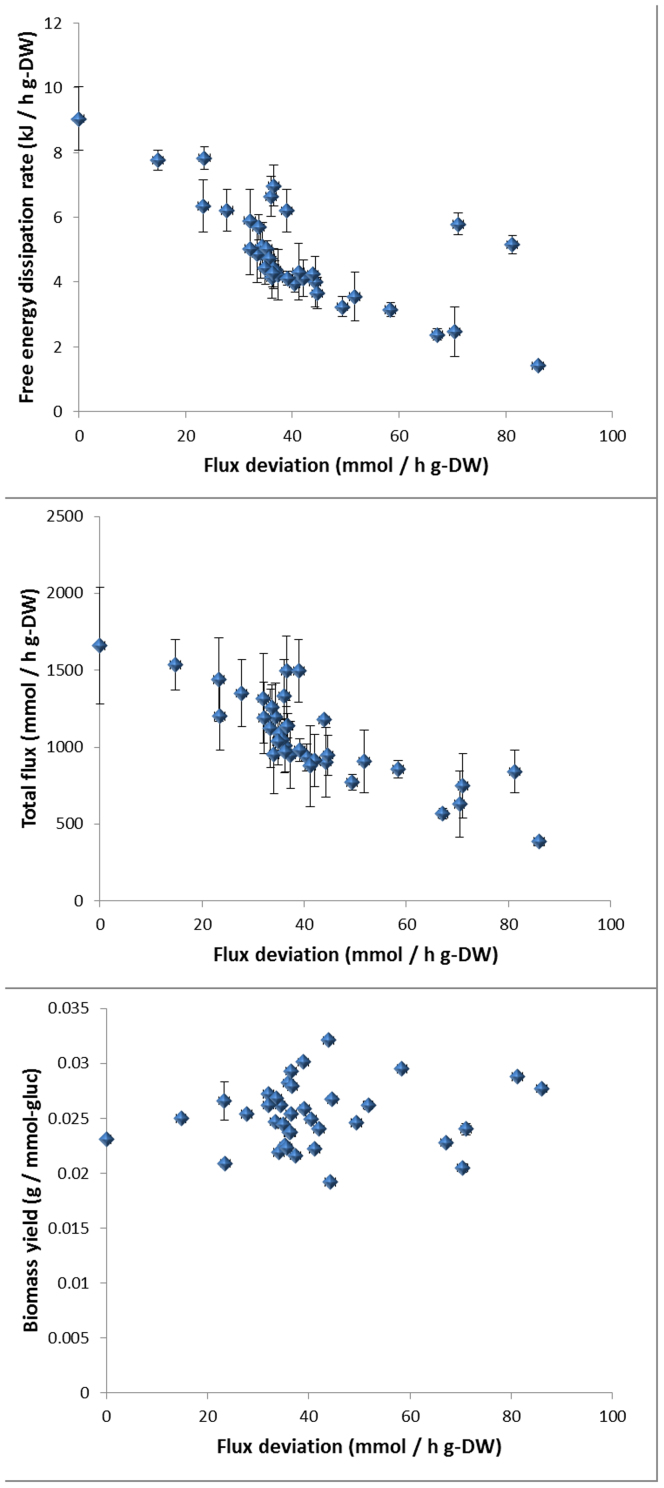
Here we plot the average value of three of the systemic functions against the Euclidean distance of the flux distributions with respect to the wild type in S. cerevisiae (this gives an idea of how these functions tend to change when the flux distributions differ more from the wild type distribution. The error bars are the standard deviations of each systemic function (calculated from the random sampling). We see that the total flux and the energy dissipation rate tend to decrease with the deviation from the wild type flux distribution, the biomass yield however takes both higher and lower values.

We also aimed to test how consistent with the experimental evidence is the assumption of the existence of an objective function maximized by the metabolic network. As we have mentioned previously the main difficulty for assessing the suitability of an objective function by comparing predictions and experimental data is the fact that besides the objective function one or several constraints must be also imposed in order to predict metabolic flux distributions. The choice of constraints determines the output of the predictions as much as the objective functions. For example it is common to constrain the carbon source uptake rate, which in reality (given the fact that it changes a lot between different conditions and as a result of gene deletions) seems to be far from being constrained. In order to avoid any ad hoc assumption of constraints, we are not comparing predictions and experiments, but the experimental values for the wild type strain with the values of deletion strains. Deletion strains have a metabolic network which is a sub-network of the wild type (and therefore has a smaller space of feasible solutions). Therefore if an objective function exists its value should be smaller or equal in the strains with a deletion than in the wild type strain. A significance test was performed (see methods) in order to identify the number of mutant strains in which each of the considered systemic variables was significantly higher or lower than in the wild type strain. The systemic variables that are not significantly higher in any of the mutant strains are possible objective functions. We see in figures 2 and 3 that the commonly used growth yield is one of the worse candidates for optimality.

**Figure 4:**
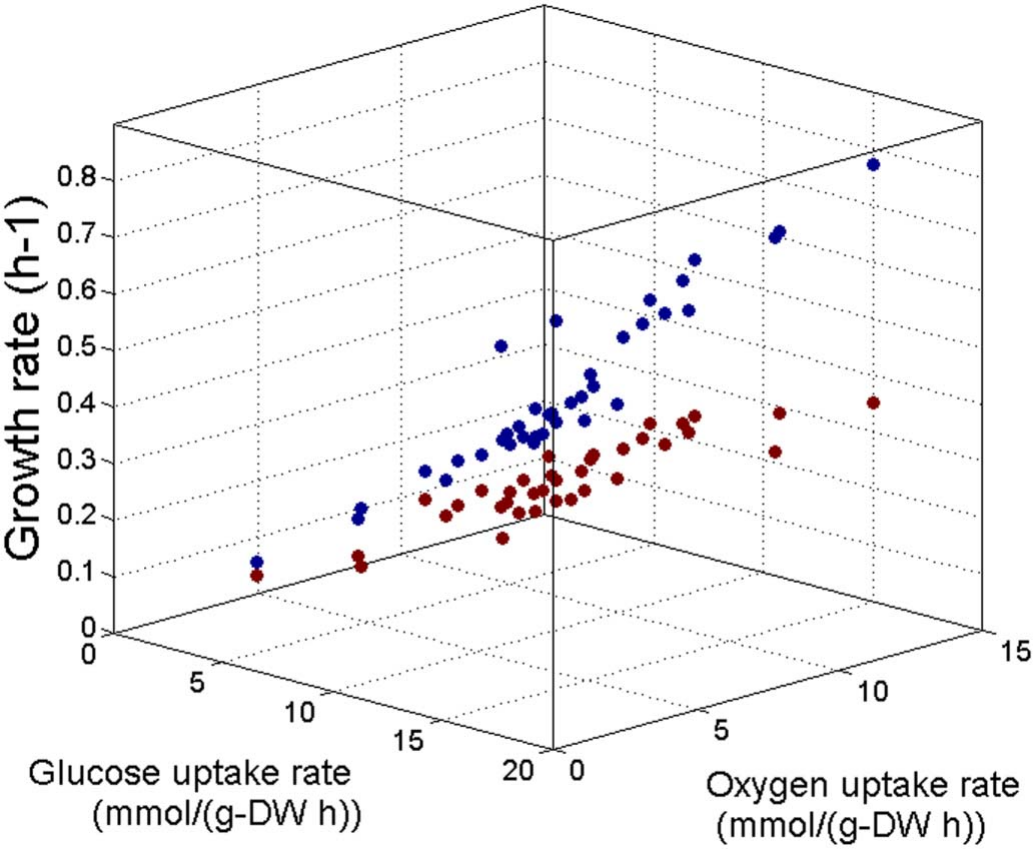
The optimal growth rate (blue dots) and the experimental growth rate (red dots) are plotted for the experimental glucose and oxygen uptake rates of the S. cerevisiae strains. The assumption of anabolic optimality strongly over-predicts the growth rate in batch conditions.

We also tested how far the biomass yields are from the assumption of anabolic optimality. By anabolic optimality we mean that the produced ATP is optimally allocated to biomass production. To test this we constrained for each strain the glucose and the oxygen consumption rates to their experimental values and compared the predicted biomass production rate with its experimental value. The real strains seemed to be well below the anabolic optimality (figure 4).

## Discussion

In summary, using two genome scale metabolic models of high quality, for a prokaryotic and a eukaryotic organism, and high quality C13 labeling experimental data^16^,^19^; we have identified three strongly conserved correlations between systemic variables. The correlation between ATP and NADH turnover rates appears to be trivial to some extent, due to the fact that both processes are highly coupled through respiration, and also fermentation. The two other correlations are non-trivial and are surprisingly well conserved for two very different microbial organisms. This points to the existence of global operation principles (involving relationships between systemic variables) of microbial metabolism that are common to eukaryotic and prokaryotic species. We also showed that if an objective function exists this is not likely to be the growth yield.

Based on our results, we suggest the utilization of the identified relationships as extra constrains in the genome-scale metabolic models. This is likely to lead to more realistic predictions of the metabolic flux distribution, at least in the two organisms we have analyzed. Using objective functions such as biomass yield does not seem to be a good option, and other objective functions (figure 2), such as total turnover of redox cofactors, are likely to be closer to reality. It has also been shown that the allocation of ATP to biosynthetic processes is clearly non-optimal in the studied strains.

## Methods

### Impossibility of a full identification of the flux distribution in a genome-scale metabolic network

A metabolic network can be described by its stoichiometric matrix *S*, which contains the stoichiometric coefficients of each metabolite (columns) in each reaction (rows) of the network. If each internal metabolite is in steady state, the following relationship must be satisfied. 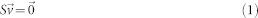
This condition defines a system with as many independent linear equations as the rank of the stoichiometric matrix and as many variables as reactions in the network. In order to solve such a system it is necessary to measure as many metabolic fluxes as the difference between the total number of reactions and the rank of the stoichiometric matrix. This would require measuring several hundreds of metabolic fluxes, which is not feasible with the current C13 labeling techniques, which typically are restricted to the fluxes in the central carbon metabolism. Therefore a complete flux distribution is today non-measurable and systemic variables such as the total ATP turnover rate or the total flux in the metabolic network are also non-measurable. This limits the conclusion of previous works^10^.

### Convex set

If the irreversibility constraints (the flux in the irreversible reactions has to be zero or positive) are added to equation 1, and the fluxes in the measured reactions are constrained to the error interval of the measurements. The sub-index i states for the irreversible reactions and the sub-index m states for the measured reactions. 
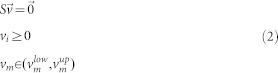
The previous set of constrains defines a convex set. A convex set *C* is defined by the following condition: if 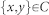 then 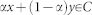 for any 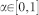. It is straightforward to see that if *x* and *y* are vectors satisfying the previous constrains, any vector defined as *αx* + *(1* − *α)y* will also satisfy the mentioned constraints.

If an element *z* of the convex set *C* can only be expressed as *z* = *αx* + *(1* − *α)y* (being *x* and *y* also elements of *C* and α a positive number lower than 1) if *z* = *x* or *z* = *y* then *z* belongs to the convex basis of *C*.

Any element *x* of *C* can be expressed as a linear combination of the elements of the convex basis of *C* {*z_i_*}, so that 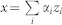 with the coefficients α_i_ being positive and their sum is equal to 1.

### Information of the distribution of coefficients α_i_

It is possible to define the Shannon's information entropy of the set of values α_i_, 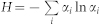. The distribution of coefficients that maximizes this information entropy corresponds to an equal value for all the coefficients. The criterion of maximal information entropy is normally assumed to describe a system of which we have a limited amount of information^21^. We use here the same criterion in order to infer a most likely flux distribution given the limited information provided by the fluxes measured experimentally. According to the mentioned criterion, if the constraints imposed in equation 2 define a convex set with a convex basis 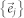 formed by *n* elements, the most likely flux distribution will be: 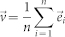.

### Random sampling of elements of the convex basis

In a genome scale metabolic network with thousands of reactions, the number of elements of the convex basis follows a combinatorial explosion that makes unfeasible its complete enumeration. It was recently shown by Kelk and co-workers, than the high number of elements in a convex basis can be explained by a small number of alternative flux values in several metabolic sub-networks, which by a mechanism of combinatorial explosion; give rise to millions of corners. Based on this property we can conclude that sampling a limited number of corners in the solution space (3000 in our case) is enough to explore all the rank of values that each reaction can take. The sampling was carried out as described in a previous paper^20^. For each sampled flux distribution we computed the value of each of the 19 systemic variables and used its average as an optimal estimator of each systemic variable for each strain.

### Comparison of systemic variables between the wild type and each mutant

We are also interested in knowing in how many mutants a systemic variable was higher or lower than in the wild type strain. To do that we compared the first sample of the wild type with all the samples of the mutant and computed how many times the mutant had a higher (or lower) value for that systemic variable. Then we repeated the same for the second sample of the wild type and continued doing the same till the last sample of the wild type. If in 95% or more of the 3000 × 3000 comparisons the mutant had a higher or lower value for the systemic variable, we considered that systemic variable as significantly higher or lower in the mutant than in the wild type.

## Author Contributions

S.B. did all the work.

## Supplementary Material

Supplementary Information S1

Supplementary Information S2
